# Effects of physical parameters on fish migration between a reservoir and its tributaries

**DOI:** 10.1038/s41598-022-12231-3

**Published:** 2022-05-23

**Authors:** Nikola Pfauserová, Marek Brabec, Ondřej Slavík, Pavel Horký, Vladimír Žlábek, Milan Hladík

**Affiliations:** 1 Department of Zoology and Fisheries, Faculty of Agrobiology, Food and Natural Resources, Czech University of Life Sciences Prague, Kamýcká 129, Suchdol, Prague 620, 165 21 Czech Republic; 2 Institute of Computer Science, The Czech Academy of Sciences, Prague, Czech Republic; 3 Faculty of Fisheries and Protection of Waters, South Bohemian Research Center of Aquaculture and Biodiversity of Hydrocenoses, University of South Bohemia in České Budějovice, České Budějovice, Czech Republic; 4 Department of Water Management Planning and Conceptions, Water Management Development and Construction Joint Stock Company (VRV a.s.), Prague, Czech Republic

**Keywords:** Animal migration, Behavioural ecology, Biodiversity, Freshwater ecology, Animal migration, Behavioural ecology, Biodiversity, Freshwater ecology, Invasive species, Invasive species

## Abstract

Reservoirs interrupt natural riverine continuity, reduce the overall diversity of the environment, and enhance the spread of non-native fish species through suitable environments. Under favourable conditions, invasive species migrate to tributaries to benefit from local resource supplies. However, the changes in physical conditions in reservoirs that motivate fish species to migrate remain poorly understood. We analysed migration between a reservoir and its tributary in three non-native (asp *Leuciscus aspius*, ide *Leuciscus idus,* and bream *Abramis brama*) and two native (chub *Squalius cephalus* and pike *Esox lucius*) species equipped with radio tags. This 5-year study revealed that an increasing day length was the most general predictor of migration into the tributary in all observed species except *E. lucius*. Only *L. aspius* responded to the substantially increasing water level in the reservoir, while the migration of *L. idus* and *S. cephalus* was attenuated. *Abramis brama* and *S. cephalus* occurred more frequently in tributaries with an increase in temperature in the reservoir and vice versa, but if the difference in temperature between the reservoir and its tributary was small, then *A. brama* did not migrate. Our results showed that migration from the reservoir mainly followed the alterations of daylight, while responses to other parameters were species specific. The interindividual heterogeneity within the species was significant and was not caused by differences in length or sex. Our results contribute to the knowledge of how reservoirs can affect the spread of non-native species that adapt to rapid human-induced environmental changes.

## Introduction

Animal migrations, fish included, can be defined as cyclic movements between two or more environments^1,2^. In temperate conditions, represented, e.g., by inland waters of Central Europe, such migrations are known for many potamodromous fish species^2,3^. Seasonal fish migrations are aimed at searching for resources such as spawning and foraging sites^4–6^, refuges^7^, and/or to manifest a nomadic lifestyle^8^. They are influenced mainly by photoperiod and/or temperature, as an increase in daylight enhances the production of reproductive hormones for the upcoming season, and an increase in temperature facilitates the movement of poikilotherm fish^2,9,10^. To allocate resources, Cyprinids migrate predominantly in spring^11,12^, whilst Salmonids upstream migrations occur mostly in summer and autumn, followed by downstream descent to refuge locations for wintering^3,13^. Apart from riverine environment, seasonal fish migrations occur between lentic and lotic environments, e.g., fish migrate from lakes to tributaries^5,6,14,15^. Similarly, cyclic fish migrations are reported between artificial reservoirs and its tributaries^6,14,16,17^.

As a result of reservoir location, dam construction type, and the ability of various species to pass obstacles, fish diversity is altered in tributaries upstream of a reservoir^18–20^. The construction of any lateral obstacle results not only in the disturbance of natural riverine connectivity^21^ but may also lead to the reduction of native species and later invasion by non-native species^22–24^ as reservoirs provide new suitable habitats for invaders^25,26^. Moreover, in artificial reservoirs, non-native species not fully acclimatized to the reservoir conditions still need to use the resources in the tributaries^14,15^, so reservoirs may facilitate their spread to riverine environments^26–28^. Hence, these species, frequently generalists, are able to benefit from the differences between the deeper environment of reservoirs with favourable conditions during the winter and, conversely, the flowing environment of rivers with a higher supply of food and reproductive sites during the spring and summer^16,29^. For example, the spring reproductive migrations of Cyprinids from the reservoir to the tributary caused a shift in community diversity and a change in the spatial distribution of native species, which moved to smaller tributaries^16,29^.

The signal for the fish to spread from the reservoir to the tributaries may be conducted by the conditions in the reservoir; e.g., low water levels in reservoirs are associated with inaccessibility to feeding and spawning sites along the shoreline of the reservoir^30^. As an increase in water levels brings new opportunities to obtain resources, it may also be considered an indication that migration could be beneficial, i.e., that there are favourable conditions in other habitats. As some species may have limited ability to overcome obstacles^31^, increasing water levels in reservoirs may signal suitable conditions for spread. However, data on what is triggering the migration of fish currently widespread in the reservoir are still very scarce.

In our study, we focused on the dynamic of fish distribution changes between reservoirs and tributaries, with the aim to describe how changes in physical conditions in the reservoir can alter the onset of fish migrations generally controlled by seasonal cycles. To achieve the aim of the study, we used two native and three non-native species that undergo seasonal migrations between the reservoir and its tributary, as reported in our previous study addressing the length of these migrations and the impact of flow conditions in the tributaries^16,29^. We used data on environmental conditions in the reservoir (e.g., temperature and water level) and a modified dataset of fish movements between the reservoir and the tributary (presence/absence in the tributary). We analysed the influence of physical conditions in the reservoir (Lipno reservoir, Czech Republic, Central Europa) on native and non-native species migrations into the tributary (Vltava River, Elbe catchment area).

## Materials and methods

### Study site and fish tagging

**Figure 1:**
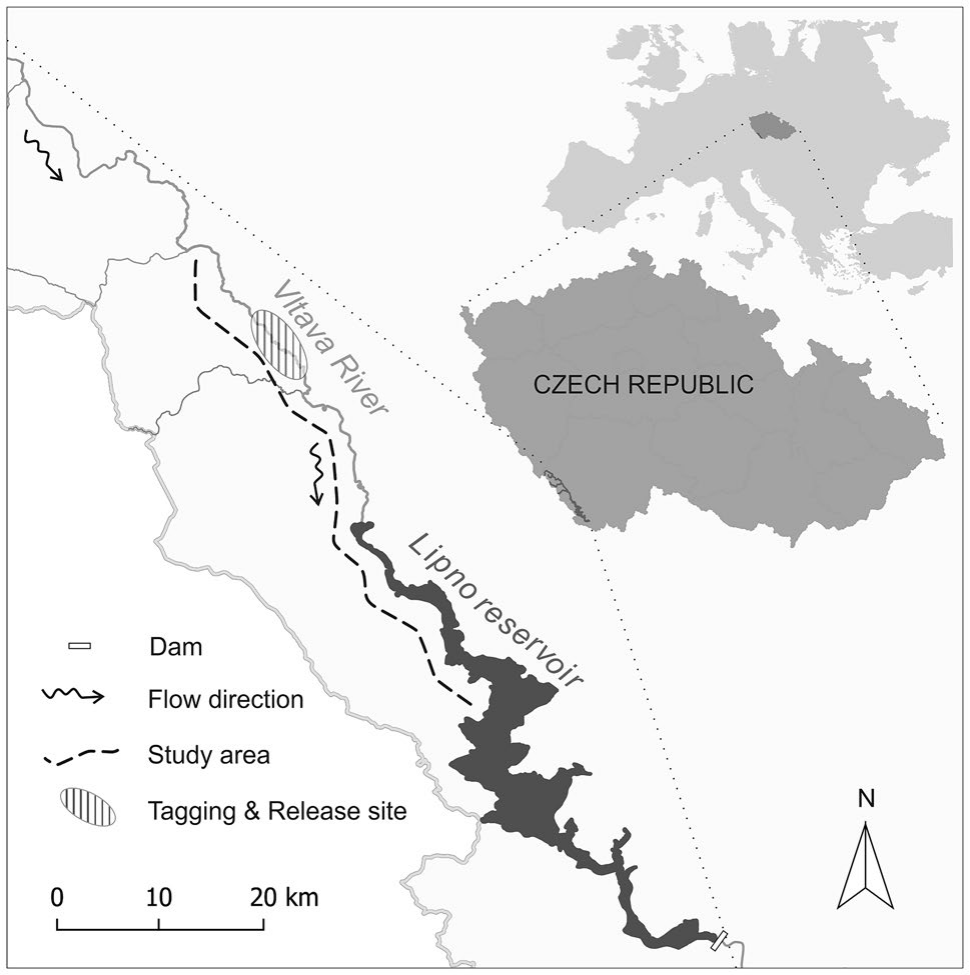
Map of the study area—the Lipno Reservoir and Vltava River. The size of the primary study area is illustrated by the dashed line (i.e., denotes the range of the study area).

Our study site was 45 km in total and was composed of a 30-km section of the free-flowing Vltava River and the upper 15 km of the Lipno Reservoir, of which the Vltava is the main tributary (Fig. 1). The majority of the study area is part of Šumava National Park, Czech Republic. The filling of the Lipno Reservoir (46.5 km^2^; maximum depth 25 m; maximum width 5 km; length 42 km) dates back to 1958. Since then, it has been used as a source of hydropower and for recreational activities, water retention, and flood protection. The farthest upstream point of occurrence of marked fish and the reservoir border the primary area where the study took place. Fish tracking was conducted only in the upper 15 km of the reservoir, as the radiotelemetry detection of transmitters at depths greater than 5 m is unstable. Regular attempts to track the fish outside the primary study area were conducted, but with a low success rate. For detailed information about the study site, see^16^.

For this study, 5 fish species were collected by electrofishing (FEG 1500, FEG 15000; EFKO-Elektrofischfanggeräte GmbH, Germany). Fish tagging was conducted during the period from May 2014 to September 2015 in the lotic segment of Vltava River in the area approx. 15–20 km upstream of Lipno reservoir (Fig. 1). All tagged fish were weighed and measured (Table 1). Under anaesthesia (using 2-phenoxy-ethanol; 0.2 mL × L^−1^), fish were equipped with radio transmitters (Lotek Engineering, Inc., Newmarket, ON, Canada); the transmitter type was chosen according to the fish weight and never exceeded 2% of the body mass of the fish^32^. Transmitters with a uniform frequency of 138.300 MHz (MCFT2-3FM, mean operational lifespan of 1432 days; NTC-6-2, 687 days; NTC-6-1, 365 days) and a uniform burst rate (5 s) were implanted into the fish body cavities through a midventral incision and secured by three separate stitches (using sterile braided absorbable sutures; Ethicon-coated Vicryl, Coated Vicryl®, Ethicon Inc., Somerville, NJ, USA). Fish were sexed during the surgery and released approximately thirty minutes later, after they had recovered their body balance and normal swimming activity.

**Table 1:** Fish used for radiotelemetry tracking.

Species	n individuals	Standard length mean (mm) ± standard error of the mean (SEM)	Body weight mean (g) ± SEM
*Abramis brama*^NNS^	47	(316.83 ± 3.71)	(631.28 ± 19.04)
*Leuciscus idus*^NNS^	29	(327.07 ± 8.98)	(769.28 ± 58.00)
*Leuciscus aspius*^NNS^	16	(542.50 ± 22.83)	(1759.69 ± 349.37)
*Squalius cephalus*	31	(371.10 ± 9.42)	(970.61 ± 70.22)
*Esox lucius*	22	(468.05 ± 24.15)	(1091.46 ± 199.48)

^NNS^Non-native species.

### Species characteristics

In total, 154 adult fish individuals of 5 species were tracked: non-native *A. brama* (L.), *L. idus* (L.), *L. aspius* (L.); and native *S. cephalus* (L.) and *E. lucius* L. (Table 1). Five *E. lucius* and four *A. brama* were caught by anglers or died during the study and therefore were excluded from the dataset. The native status of species to the upper stretches of the Vltava River was considered according to the assemblage composition before filling the Lipno Reservoir^33^. We refer to the species whose occurrence in the Vltava River was related to the influence of the Lipno Reservoir as non-native (i.e., their presence is recorded only after reservoir construction).

*Abramis brama* is an insectivorous, phytolithophilous, eurytopic Cyprinid species^34,35^ that occurs in the whole longitudinal profile of rivers from an estuary into the sea to medium sized streams including their flood plain areas^36–38^, and also thrives in reservoirs^17,39^. Although *A. brama* shows homing and maintains stable home ranges, its ability to spread across various habitats and variability in food availability can be considered reasons for the species nomadism^8^. Accordingly, the species undertakes various intensive long-distance migrations^40,41^, reflecting the availability of different habitats^38,42^ and seasonal variability in temperature, flow, and tide phase^42,43^. Seasonal changes in spatial distribution can be altered by the predation^4,44^ and/or behavioural patterns of local subpopulations^42^. *Leuciscus idus* is an insectivorous phytolithophilous, benthopelagic, eurytopic Cyprinid species^34,35^ that undergoes long-distance migrations of tens or hundreds of km and occupies various habitats in rivers, lakes, and reservoirs^45–48^. The species displays the potential to be invasive^34,45,49^, and its migrations from a reservoir into tributaries occurred mostly in spring, although some individuals stayed in the riverine environment across the whole season^16^. *Leuciscus aspius* is a large, visually-oriented predator, lithophilous and rheophilic Cyprinid species^34,35^ that occupies lentic and lotic environments^17,50–52^ and is also referred to as an invasive species^53^. In riverine environments, movement activity predominantly occurs during summer to maximize food intake^50,54^. The species migrates from reservoirs to tributaries to exploit reproductive and feeding resources and can remain there from spring to autumn^51,52^. *Squalius cephalus* is an omnivorous, lithophilous and rheophilic Cyprinid species^34,35^ that occupies lentic and lotic environments^17,55,56^. In riverine environments, the species undertakes migrations aimed at finding spawning areas in spring and summer and/or wintering refuges, including local migrations, to find suitable habitats^2,55,56^. In reservoirs, the species primarily occupies the inflow area and often migrates into the tributaries^17^. Seasonal cyclic migrations of *S. cephalus* can be altered, e.g., by flow and/or parasites, and the primary motivation of its migrations from reservoirs into the tributaries seems to be the allocation of feeding and reproductive resources^16,56^. *Esox luciu*s is a phytophilous, eurytopic species^34,35^ that occupies rivers and/or lakes and reservoirs^57^. Its occurrence as a sit-and-wait predator is tied to submerged vegetation, while open water and pelagial areas are avoided. Intensive seasonal migrations correspond with increasing temperature and spawning period^58,59^. The species exhibits stationary and/or migratory behaviour in both environments^59,60^, and migrations from the reservoirs to tributaries to search feeding and reproductive resources were recorded^16,57–59^.

### Animal tracking and physical parameters

For five consecutive years (May 2014–December 2018), the primary study area was surveyed by boat every 14 days, on average. The frequency of surveying was higher during spring (February to April), i.e., weekly at a minimum. In contrast, during winter, tracking was conducted at least twice in one month due to harsh winter conditions, making it impossible to use the boat for tracking (substituted by walking). Tracking by walking was performed in only a few cases during wintertime, when the reservoir surface was frozen; the tracking trace was always along the same trail (the river streamline above the original riverbed following the usual trail of the boat). The river was navigable throughout the season. The tracking equipment was composed of a radio receiver (Lotek SRX_600; Lotek Engineering Inc., Ontario, Canada) and a three-element Yagi antenna. Each tracking event was composed of a one-way survey downstream with continuous recording of individual fish positions. The fish positions were stored in a global positioning system (GPS) device (GPS map 76S, Garmin LTD., USA).

**Figure 2:**
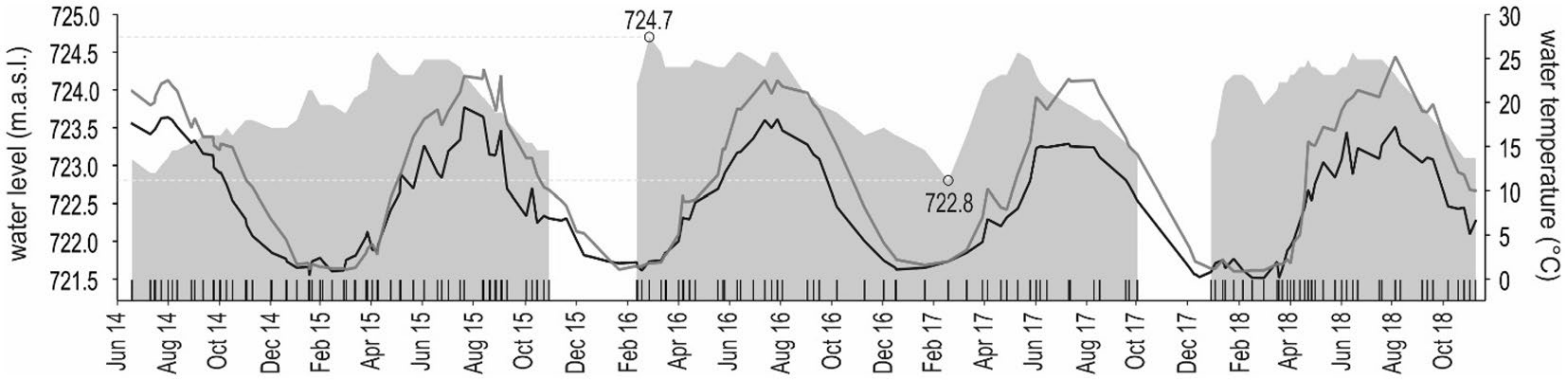
Dynamics of water levels (m.a.s.l.) in the Lipno Reservoir (grey area) with maximum and minimum values marked by circles, and temperatures (°C) in the Lipno Reservoir (grey line) and the Vltava River (black line) in the years 2014–2018. The spaces between grey areas represent periods with no available water level data for technical reasons.

Light is a complex external and ecological factor whose components include the colour spectrum (quality), intensity (quantity) and photoperiod (periodicity). In our study, we focused on the periodic aspect of light supply, i.e., daylength, and we referred to this factor as the photoperiod^61^. Temperature in the Lipno Reservoir and its tributary, the Vltava River, was measured by Povodí Vltavy, State Enterprise (www.pvl.cz/en; Fig. 2). The water level (mean 723.86 m.a.s.l., range 722.8–724.7 m.a.s.l.; Fig. 2) was recorded at the Lipno Reservoir by Povodí Vltavy. The long-term data were collected and stored by project Hydra^2^ on the website http://hydra2.dusanrysavy.cz. Data created or used during this study are openly available online^62^.

Data were collected in accordance with the Guide for the Care and Use of Animals of the Czech University of Life Sciences Prague and all experimental protocols were approved by the Faculty of Agrobiology, Food and Natural Resources Licensing Committee (Expert Commission Ensuring Welfare of Experimental Animals). All of the experimental procedures complied with valid legislative regulations (Law no. 246/1992, §19, art. 1, letter c), which were derived from the Directive 2010/63/EU; additionally, the permit from the Ministry of Environment of the Czech Republic (no. 53139/ENV/14-3074/630/14) was subjected to O. Slavík, qualified according to Law no. 246/1992, §17, art. 1; permit no. CZ00167. All sampling procedures were carried out with the relevant permissions from the Departmental Expert Committee for Authorization of Experimental Projects of the Ministry of Agriculture of the Czech Republic (permit no. 88042/2014-MZE-17214) in compliance with EU legislation. The study was in compliance with ARRIVE guidelines. The internal licensing committee gave ethical approval for the same project documentation as the Ministry of Environment of the Czech Republic, i.e., approved project no. 53139/ENV/14-3074/630/14.

### Statistical analyses

We analysed data on the presence of fish in the tributary via a flexible semiparametric logistic regression model that can be perceived either as a GLMM (generalized linear mixed model^63^) or, more broadly, as a GAM (generalized additive model^64,65^) We accounted for autocorrelation induced by repeated observations of the same individual by the inclusion of random individual effects. For potentially nonlinear effects, even on the logistic scale, we used nonparametric terms. These were implemented as complexity-penalized splines^66^ with quadratic penalties^65^. Unknown penalty coefficients were estimated via UBRE (unbiased risk estimator^65^). Given the penalties, the model parameters and nonparametric coefficients were estimated simultaneously via maximization of the penalized likelihood^67^. All computations were performed in the R computing environment^68^ and with the mgcv package^65^.

Our model used for testing the effect of photoperiod, the difference in temperature between the reservoir and the tributary and the water level in the reservoir on the probability of fish presence in the tributary (while correcting for the effects of daylight length, reservoir versus tributary temperature difference, study year, fish sex, fish length and random individual effect) was as follows:

1 $$\begin{aligned} Y_{ti} \sim & Binomial\left( {\pi_{ti} ,1} \right) \\ log\left( {\frac{{\pi_{ti} }}{{1 - \pi_{ti} }}} \right) = & \beta_{0} + b_{i} + \beta \cdot \left( {{\text{individual}} \,i\, {\text{is a male}}} \right) + \mathop \sum \limits_{r} \alpha_{r} \cdot I\left( {{\text{time}}\, t\, {\text{is in year}} r} \right) + s_{l} \left( {length_{i} } \right) \\ & + s_{daylength} \left( {daylength_{t} } \right) + s_{difT} \left( {Tres_{t} - Ttrib_{t} } \right) + s_{level} \left( {water \,level_{t} } \right). \\ \end{aligned}$$

The individual model terms are listed in Table 2 and discussed in the following sections.

**Table 2:** Parameters used in the model for testing the effect of water level.

Parameter	Description
${Y}_{ti}$	Observed indicator of fish presence in tributaries (1 if the *i*-th fish was present in the tributaries at time t, and 0 if it was present in the Lipno reservoir at time t)
$log\left(\frac{x}{1-x}\right)$	Logit transformation (canonical link for the binomial distribution)
${\beta }_{0}$	(Unknown) intercept
${b}_{i}$	The random effect of the *i*-th individual fish. We assume Gaussian distribution for values of b and that they are distributed independently across individuals
$I\left(.\right)$	Indicator function (assumes the value of 1 if its argument is true and 0 otherwise)
β	Effect of a male individual (allowing for sex-specific marginal effect upon probability of occurrence in the river)
${\alpha }_{r}$’s	Coefficients allowing for different presence in different calendar years in which the study was conducted
${s}_{level}$	Unknown smooth, potentially nonlinear, function implemented as a cubic spline of main interest to be estimated from data
${s}_{l}$	Smooth effect of fish length to be estimated from data
${s}_{difT}$	Smooth effect of the water temperature difference (between the reservoir and tributary, on the day corresponding to observation time *t*) to be estimated from data
${s}_{daylength}$	Smooth effect of photoperiod (measured in hours)

The whole $\sum_{r}{\alpha }_{r}\cdot I\left(time\, t \,is\, in \,year\, r\right)$ term is an ANOVA-like component adjusting for possible differences in tributary presence probability among the years. Generally, identifiability considerations require one restriction on the coefficients. We used the standard baseline (or “treatment” parameterization in R terminology) restriction ${\alpha }_{2014}=0$.

To address the problem of autocorrelation among repeated observations of the same individual^69^ and pseudoreplication, as one of its consequences^70^, we used individual random effects in our models accounting for interindividual heterogeneity. The net effect of using this comprehensive model is that we can correct the water level term to adjust for nuisance terms and random individual variability. As a result, we can isolate, test, and depict the effects of the correcting terms and evaluate the variability connected with the random individual effects. We present p value for all model terms (Table 3).

**Table 3:** P value for individual components of the model (1).

	*A. brama*^NNS^	*L. idus*^NNS^	*L. aspius*^NNS^	*E. lucius*	*S. cephalus*
Year	1.000	< 0.001***	0.237	0.887	< 0.05*
Sex	1.000	0.699	0.1	0.969	0.975
Random ind. effect	< 0.001***	< 0.001***	0.235	0.867	< 0.001***
Photoperiod	< 0.001***	< 0.001***	< 0.05*	0.994	< 0.001***
Length	0.657	0.986	0.06	1.000	0.926
Temperature	< 0.05*	< 0.169	0.359	0.989	< 0.001***
Water level	0.114	< 0.001***	< 0.05*	0.998	< 0.05*

^NNS^Non-native species.

*P ≤ 0.05; ***P ≤ 0.001.

## Results

### Effect of photoperiod

**Figure 3:**
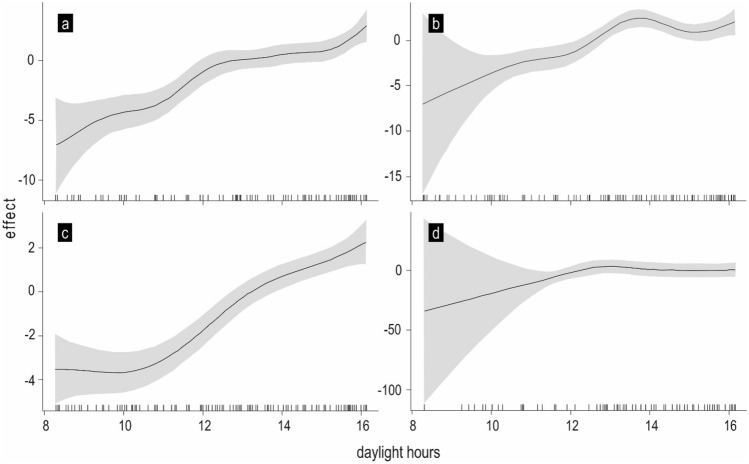
Effect of photoperiod on the probability of *A. brama* (**a**), *L. idus* (**b**), *S. cephalus* (**c**) and *L. aspius* (**d**) occurrence in the tributary. The solid line is an estimate of s_daylength_; the shaded region shows (pointwise constructed) 95% confidence intervals for a given photoperiod.

Photoperiod significantly influenced all non-native species and native *S. cephalus* (Table 3). The probability of occurrence in the tributary in these species increased with a prolonged photoperiod, being the highest at the beginning of the process and flattening later, as apparent mainly for *L. idus* and *L. aspius*. An increasing photoperiod was the signal for native and non-native species in the reservoir to migrate into the tributary (Fig. 3).

### Effect of the difference between temperatures in the reservoir and its tributary

**Figure 4:**
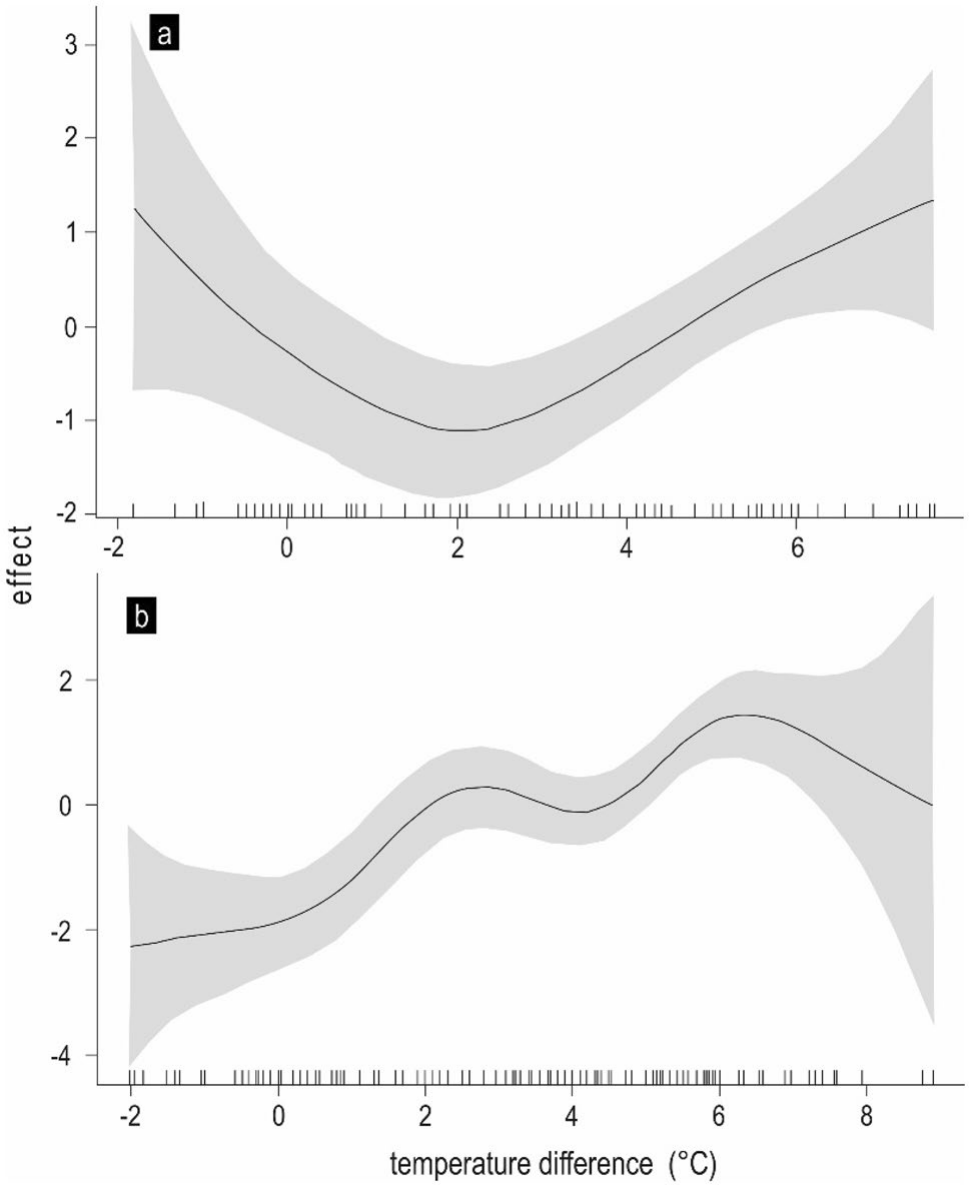
Effect of differences between temperatures in the Lino Reservoir and its tributary, the Vltava River, on the probability of *A. brama* (**a**) and *S. cephalus* (**b**) occurrence in the tributary. The solid line is an estimate of s_difT_; the shaded region shows (pointwise constructed) 95% confidence intervals for given temperature difference values.

The difference in temperatures between the reservoir and its tributary significantly influenced the non-native *A. brama* and native *S. cephalus* (Table 3). The lowest probability of *A. brama* occurrence in the tributary was recorded when the difference between the temperature of the reservoir and its tributary was small (Fig. 4a). If the temperature in the reservoir was lower than (negative difference in temperatures) and/or comparable to the temperature of the tributary, then the probability of migration from the reservoir into the tributary decreased and vice versa. Similarly, the probability of migration of *S. cephalus* from the reservoir increased with a larger difference between the temperatures in the reservoir and its tributary, but it started to decrease when the temperature in the reservoir reached its maximum (Fig. 4b). Higher temperatures in the reservoir were a signal for migration into the tributary for both native and non-native species.

### Effect of water level

**Figure 5:**
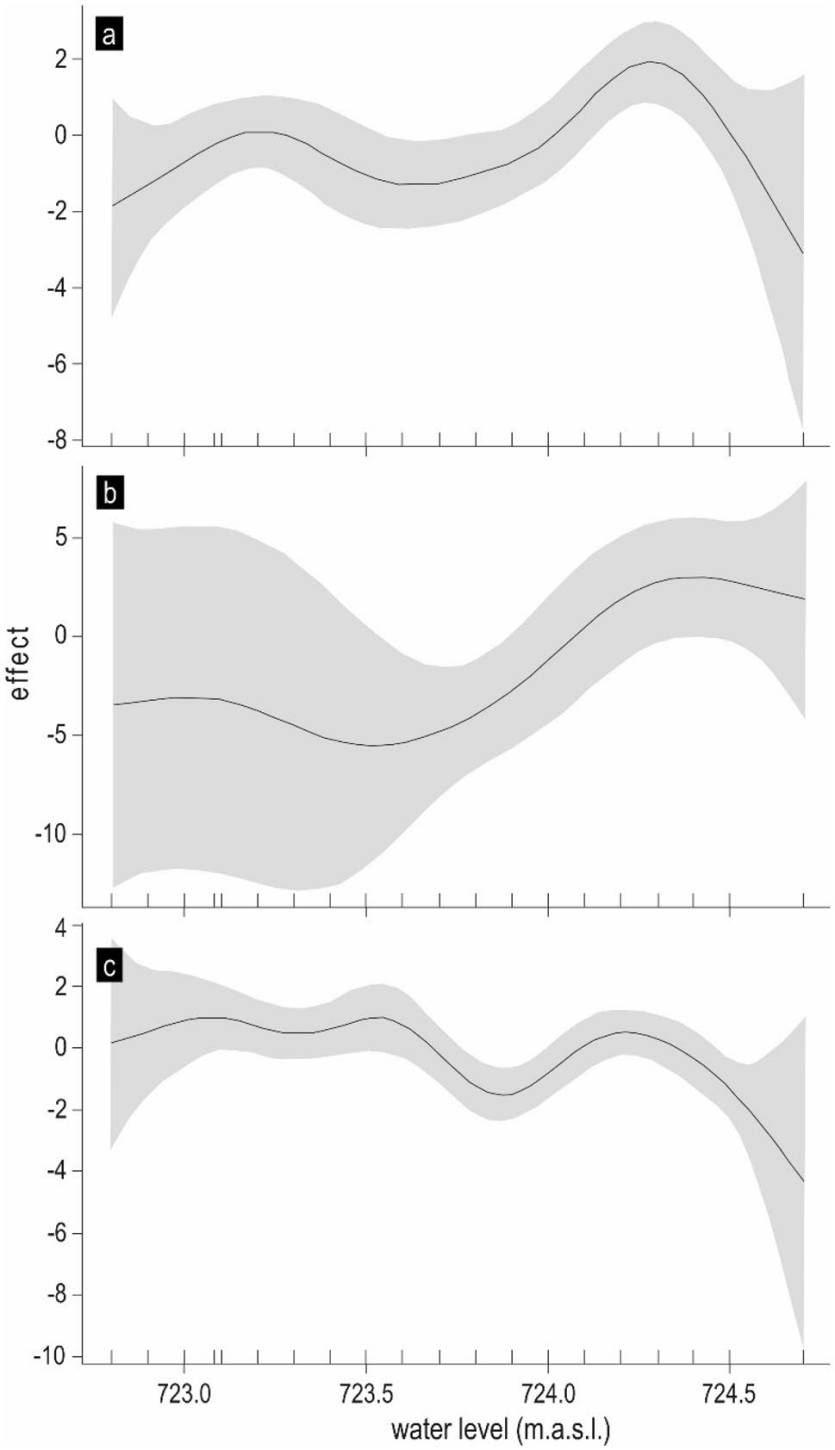
Effect of water level on the probability of *L. idus* (**a**), *L. aspius* (**b**) and *S. cephalus* (**c**) occurrence in the tributary. The solid line is an estimate of s_level_; the shaded region shows (pointwise constructed) 95% confidence intervals for given water level values.

The water level effect was significant for non-native *L. idus* and *L. aspius* and for the native species *S. cephalus* (Table 3). For *L. idus*, there was a clear negative effect of extremely high water levels and a positive effect of extremely low water levels on the probability of this species being in the tributary (Fig. 5a). The large plateau between these extremes shows that most typical water levels did not appreciably change the probability. Mildly ascending values of water levels did not affect the migration of *L. aspius* into the tributary, while substantial water level ascent was followed by a significantly increasing probability of tributary occurrence until water levels reached the maximum when the effect dissipated (Table 3, Fig. 5b). For *S. cephalus*, a considerable effect of water level was recorded as a decrease in the probability of species occurrence in the tributary in accordance with the maximal water levels in the reservoir (Table 3, Fig. 5c). The effect of water level was species specific, and only for non-native invading *L. aspius* was a positive relationship between water level ascent and the probability of species occurrence in the tributary observed.

### Effects of individual variability, length, sex, and observation period

Individual variability contributed to migration between the reservoir and the tributary for *A. brama*, *L. idus,* and *S. cephalus*; however, this variability was not due to the length of the individuals, since these variable effects were not significant for any of the observed species. Moreover, for *L. idus* and *S. cephalus,* there were significant differences among the study years in the probability of tributary occurrence. Sex was not significant for any of the observed species. *Leuciscus aspius* migration was consistent and not affected by intraindividual variability. For native *E. lucius*, the effect of individual variability was not significant (Table 3).

## Discussion

Our study investigated the differences in the behaviour of native and non-native fish species by analysing the relationship between their migration and variability in environmental conditions in the reservoir. Our results revealed that both natives and non-natives responded to changes in physical conditions by migration between the reservoir and its tributary. One of the two natives, *S. cephalus*, responded to all observed parameters, while for the migration of *E. lucius*, no impact of the observed parameters was identified, although the species has been found to migrate between riverine and reservoir habitats^57^. One potential reason for this result is the availability of sufficient resources, e.g., those for reproduction, feeding and sheltering^58,59^, for *E. lucius* in both the reservoir and tributary habitats, causing the movement of the species between the two habitats to be random.

In our study, a prolonged photoperiod was the most common parameter affecting migration from the reservoir into the tributary for both natives and non-natives. The synchronization of physiology and behaviour according to day length is a general predictor of freshwater fish migration regardless of the type of environment^2^. A prolonged photoperiod is a signal for upcoming reproduction for species that spawn in spring and early summer (all observed species in our study)^71,72^. For example, the migration of *S. cephalus* through fish ladders was significantly correlated with a prolonged photoperiod, demonstrating an escalating effort of fish to reach spawning sites^73,74^. For non-natives, changes in parameters in the reservoir were associated with migration into the tributary; two non-natives responded to water level changes, while one species responded to temperature. Our results suggested that fish migration from the reservoir into the tributaries was highly species specific and that changes in the environmental parameters of the reservoir played a key role in motivating fish to initiate their migration. In contrast, migration within the river and migration length are determined by the inner conditions in the river, e.g., flow^16^. Only non-native *A. brama* and native *S. cephalus* were impacted by the difference in temperatures between the two observed habitats in terms of migration from the reservoir into the tributary. Mainly, the responsiveness of *A. brama* was high, showing negligible migration between the reservoir and the tributary when the difference in temperatures between the two habitats was low, while an increase in temperature in the reservoir was accompanied by a significant increase in its migration into the tributary and vice versa. *Abramis brama* intensively migrates within the longitudinal profile of main rivers^41,43^ and enters tributaries with shallow and warmer waters^43^. Interestingly, in our study, individuals occupying the reservoir used an increase in temperature as a signal for migration into the tributary, while increased temperature in the tributary elicited no response. This result was likely due to the large scale of the reservoir, 42 km in length, in which broadly dispersed individuals of the species are unlikely to have contact with the environment of the tributary. Moreover, the studied stream, the Vltava River, the main tributary of the reservoir, contains significantly colder water than the Lipno reservoir throughout much of the year; hence, its temperature does not appear to be a signal for migration of the species. *Abramis brama,* which inhabits a wide range of habitats and often occurs in slow-flowing rivers or shallow lakes^72,75^, showed partial migration explained, e.g., by dependence on predation risk^44^. *Abramis brama*, an opportunistic phytolithophilic species^34^, utilizes lentic habitats for spawning, as a nursery, and for flow refuge^40,43^. The fact that *A. brama,* as a spawning generalist, utilizes both tributaries and reservoirs for its reproduction may explain its weak response to changes in the water level but significant response to temperature changes. In contrast, in our study, native *S. cephalus* also migrated from the reservoir into the tributary when the water in the reservoir was colder than that in the tributary. As reported in many studies, reservoirs facilitate the spread of species into streams^27,28,76–78^. Although photoperiod is the most influential factor, it appears that an increasing temperature in the Lipno Reservoir was an important factor affecting the temperature-dependent spread of non-native fish species from the reservoir.

Only highly invasive *L*. *aspius* responded to the rising water level in the reservoir. The relationship between water level changes and *L. aspius* migration from the reservoir into the tributary did not show any linear change over time, as the species migrated only in response to a significant increase in the water level. *Leuciscus aspius* are benthopelagic, rheophilic, potamodromous predators^50,54,72^ known to migrate upstream from reservoirs and utilize tributary resources for prolonged periods, possibly suppressing native species^29,52,79^. Colonizing *L. aspius*, e.g., in France^80^ and Spain^53^, has great invasive potential due to its migratory behaviour^29,50,79^ supported by predation^72^. The upstream movement of *L. aspius* is driven mainly by spawning migration with a lithophilic preference for gravel substratum^34,72^ and by migration to feeding grounds in rivers since the species predominantly prefers lotic environments^35^. For the non-native species *L. idus*, migration from the reservoir into the tributary was influenced by extreme water levels in the reservoir; low water levels induced species migration into the tributary, while migration decreased when maximal water levels were present. *Leuciscus idus* is a benthopelagic, rheophilic, potamodromous, omnivorous fish occupying a wide range of habitats, including rivers, lakes, and even brackish waters, which facilitates its spread to new environments and may hence pose a threat to native assemblages^34,45,49^. Long migrations and behavioural flexibility in response to variation in environmental conditions have been observed and indicate the potential for this species to become an invader^46–48^. As an inconspicuous invader, *L. idus* has already shown the potential to invade new waters since it was introduced into the United Kingdom, France^81^, the Netherlands, New Zealand^82^, and the USA^83^. When migrating upstream, *L. idus* resides in areas directly downstream of obstacles^47^ and has also shown behaviour consistent with waiting for suitable conditions, e.g., an increase in the water level to pass an obstacle^84^. This suggests that despite its long-distance migratory potential, the species avoids high velocities and does not pass obstacles by jumping, being native in downstream stretches of large rivers^71,72^. Similarly, *S. cephalus* responded to an increase in water level by a decrease in migration into the tributary. Although the native fish *S. cephalus* is a lithophilic, rheophilic species^34,35^, it often uses the reservoir as an environment for maturing and spawning, where its populations thrive^17^. Hence, an increase in the water level in the reservoir could serve as a signal to inhabit submerged habitats within the reservoir. Only *L. idus* and *S. cephalus* showed a significant influence of variability over the study period on their occurrence in the tributary; hence, we infer a complex impact of parameters involving the relationship between the water level in the reservoir and temperature and its variability over the years.

Our results revealed no influence of sex or body length on the presence of fish in the tributary. Sex plays an important role in the timing of migrations if spawning areas are approached by each sex at different times according to their roles at the site, e.g., site selection, defence and use for spawning^2^. However, all the cyprinid species examined in our study are promiscuous spawners for which an influence of sex on migration is not expected, and similarly, the influence of sex is not expected in space- and/or diet-motivated migrations. Fish body size plays an important role in migrations between lentic and lotic environments, particularly for fish that are prey^44,85,86^. Our results were probably affected by the telemetry method used, being selective in tagging fish of a certain size that corresponded with the long-life radio tags and a necessary minimal ratio between the weight of the fish and the tag. Nevertheless, individual variability in fish distribution between the reservoir and the tributary occurred for the majority of the observed species, probably reflecting differences in the propensity of an individual to migrate^87–89^.

In conclusion, the migration of both, native and non-native species between the reservoir and its tributary was influenced mainly by the photoperiod. Our study, however, revealed an alteration in the migration scenario for non-natives in response to temperature and water level changes in the reservoir, suggesting the potential for water management measures to avoid invasive species spread into a new environment^89–92^, hence supporting the protection of native assemblages in the tributaries. The manipulation of the water level in the reservoir and/or the installation of suitable lateral obstacles at the tributaries should be considered to avoid the upstream migration of invaders from the reservoir into the upstream stretches of the tributaries.

## Data Availability

The dataset generated and analysed during the current study is available in the Mendeley Data repository^62^.
